# The Impact of Vitamin D Deficiency on Depression: A Systematic Review

**DOI:** 10.1192/j.eurpsy.2026.11387

**Published:** 2026-07-31

**Authors:** M. F. Zeilstra, M. Arts, K. M. Zeilstra-Kalt, L. de Jonge

**Affiliations:** 1 Integrative Medicine, Center de Wetering, Leeuwarden; 2 Department of Geriatric Psychiatry and Neuropsychiatry, GGZ-WNB, Halsteren; 3 Integrative Medicine, Leonardo Scientific Research Institute, Groningen, Netherlands

## Abstract

**Introduction:**

Vitamin D deficiency is increasingly recognized as a risk factor for depression. It plays a key role in both immune regulation and brain function. This systematic review synthesizes evidence on the link between low vitamin D levels and depressive symptoms. Findings consistently show that deficiency is associated with higher prevalence and severity of depression. Supplementation appears to reduce symptoms, especially in deficient individuals. Proposed mechanisms involve modulation of neuroinflammation and regulation of neurotransmitters. These results highlight the importance of vitamin D status for mental health and future research directions.

**Objectives:**

The aims of this review are to: (a) summarize evidence on the association between vitamin D deficiency and depression, (b) assess whether supplementation improves depressive symptoms in adults, and (c) explore potential biological mechanisms underlying this link.

**Methods:**

A systematic search of PubMed, EMBASE, and additional databases was conducted. Only randomized controlled trials examining serum 25(OH)D concentrations in relation to depression were included.

**Results:**

Low vitamin D levels were linked to a higher risk of depression. Supplementation led to significant improvement in depression scores, with the strongest effects observed in participants who were deficient at baseline and who received doses above 2,000 IU/day. Potential mechanisms include impaired synthesis of serotonin and dopamine, reduced expression of neurotrophic factors such as BDNF, increased neuroinflammation driven by pro-inflammatory cytokines, and disrupted calcium homeostasis leading to excitotoxicity. Together, these alterations may affect brain networks involved in cognition and emotional regulation.

**Conclusions:**

Evidence consistently shows that vitamin D deficiency increases the risk of depression and that supplementation provides a clinically meaningful reduction in symptoms. Vitamin D may serve as an effective adjunctive therapy, particularly for individuals with low serum levels. Further research is needed to determine optimal dosage and treatment duration.

**Disclosure of Interest:**

None Declared

